# Hypertrophy and/or Hyperplasia: Dynamics of Adipose Tissue Growth

**DOI:** 10.1371/journal.pcbi.1000324

**Published:** 2009-03-27

**Authors:** Junghyo Jo, Oksana Gavrilova, Stephanie Pack, William Jou, Shawn Mullen, Anne E. Sumner, Samuel W. Cushman, Vipul Periwal

**Affiliations:** 1 Laboratory of Biological Modeling, National Institute of Diabetes and Digestive and Kidney Diseases, National Institutes of Health, Bethedsa, Maryland, United States of America; 2 Mouse Metabolism Core Laboratory, National Institute of Diabetes and Digestive and Kidney Diseases, National Institutes of Health, Bethedsa, Maryland, United States of America; 3 GPP/OITE/OIR/OD, National Institutes of Health, Bethesda, Maryland, United States of America; 4 Clinical Endocrinology Branch, National Institute of Diabetes and Digestive and Kidney Diseases, National Institutes of Health, Bethedsa, Maryland, United States of America; 5 Diabetes Branch, National Institute of Diabetes and Digestive and Kidney Diseases, National Institutes of Health, Bethedsa, Maryland, United States of America; University of Virginia, United States of America

## Abstract

Adipose tissue grows by two mechanisms: hyperplasia (cell number increase) and hypertrophy (cell size increase). Genetics and diet affect the relative contributions of these two mechanisms to the growth of adipose tissue in obesity. In this study, the size distributions of epididymal adipose cells from two mouse strains, obesity-resistant FVB/N and obesity-prone C57BL/6, were measured after 2, 4, and 12 weeks under regular and high-fat feeding conditions. The total cell number in the epididymal fat pad was estimated from the fat pad mass and the normalized cell-size distribution. The cell number and volume-weighted mean cell size increase as a function of fat pad mass. To address adipose tissue growth precisely, we developed a mathematical model describing the evolution of the adipose cell-size distributions as a function of the increasing fat pad mass, instead of the increasing chronological time. Our model describes the recruitment of new adipose cells and their subsequent development in different strains, and with different diet regimens, with common mechanisms, but with diet- and genetics-dependent model parameters. Compared to the FVB/N strain, the C57BL/6 strain has greater recruitment of small adipose cells. Hyperplasia is enhanced by high-fat diet in a strain-dependent way, suggesting a synergistic interaction between genetics and diet. Moreover, high-fat feeding increases the rate of adipose cell size growth, independent of strain, reflecting the increase in calories requiring storage. Additionally, high-fat diet leads to a dramatic spreading of the size distribution of adipose cells in both strains; this implies an increase in size fluctuations of adipose cells through lipid turnover.

## Author summary

Obesity is an enlargement of adipose tissue to store excess energy intake. Hyperplasia (cell number increase) and hypertrophy (cell size increase) are two possible growth mechanisms. The *in vivo* dynamic change of fat tissue cannot be monitored in real time due to current technical limitations. However, we can measure cell-size distributions of fat cells in individual animals. Our fundamental goal is to extract dynamic features of tissue remodeling from snapshots of cell-size distributions. We develop a mathematical model that interpolates between the cell-size distribution measurements and predicts the continuous change of the cell-size distribution with respect to fat pad mass increase. Our adipose tissue growth model includes three essential components: new cell recruitment, size-dependent cell growth, and cell-size fluctuations. In particular, we compared the adipose tissue growth of obesity-prone and obesity-resistant mice under a standard or a high-fat diet to examine the genetic and diet effect on adipose tissue growth. By applying our model to these different conditions, we found that the size increase of fat cells is dependent on diet. On the other hand, the diet-induced number increase of fat cells is dependent on strain, suggesting a synergy between genetics and diet.

## Introduction

Obesity is an enlargement of adipose tissue to store excess energy intake. Hyperplasia (cell number increase) and hypertrophy (cell size increase) are two possible growth mechanisms. Adipose tissue obesity phenotypes are influenced by diet and genetics, as well as by their interaction [1]–[4]. Starting from Johnson and Hirsch's studies [5], there is an extensive literature on adipose tissue growth in normal and abnormal development, characterizing the state of the tissue in terms of the mean cell size and cell number. Hyperplastic growth appears only at early stages in adipose tissue development [6],[7]. Hypertrophy occurs prior to hyperplasia to meet the need for additional fat storage capacity in the progression of obesity [8]. However, it has proven difficult to understand how diet and genetics specifically affect hyperplasia and/or hypertrophy of adipose cells, because of limited longitudinal data about adipose tissue growth.

Beyond these studies, it has recently become possible to measure cell-size distributions precisely. This detailed information, compared with the mean cell size and total cell number, can be used to compute many size-related quantities that permit a finer characterization of the adipose tissue growth process. Cumulants of the cell-size distribution can be used to compute other physiological quantities such as the volume-weighted mean cell size. The cell-size distribution can be used to estimate total cell number within a fat pad from its mass. Furthermore, it is believed that some specific metabolic properties, e.g., insulin resistance [9] and adipokine secretion [10], depend on the precise cell-size distribution rather than the mean cell size. Indeed, several studies have addressed the change of the size distribution of adipose cells under various conditions in chick embryo development [11], lean and obese Zucker rats [12],[13], partially lipectomized Wistar rats [14], rabbit biopsy [15], and human adipose tissue [16],[17]. These studies focused only on the static differences between cell-size distributions under different conditions. However, cross-sectional static cell-size distributions for a range of snapshots of animal development can be used to deduce the dynamics of adipose tissue growth, if we can appropriately analyze the snapshots with the help of mathematical modeling. Given present technical limitations, this may be the best available approach to a microscopic and longitudinal understanding of *in vivo* adipose tissue growth, although a recent experiment has obtained microscopic observations of lipid accumulation in lipid droplets of adipose cells [18].

To address genetic and dietary effects on the dynamic process of adipose tissue growth, we obtained cell-size distributions of epididymal fat of obesity-resistant FVB/N (hereafter FVB) and obesity-prone C57BL/6 (C57) mouse strains under standard chow and high-fat diets. The C57 mouse is the best characterized model of diet-induced obesity [19], and the FVB mouse is a preferable model for generating transgenic mice [20]. These two commonly-used inbred mouse strains are genetically quite distant [21],[22], and they have distinct metabolic phenotypes: Compared with FVB mice, C57 mice have low circulating triglyceride levels [21] and increased triglyceride clearance [23],[24]; FVB mice are characterized by relatively higher hepatic insulin resistance, counter-regulatory response to hypoglycemia, and reduced glucose-stimulated insulin secretion [25]; FVB mice are also known to be spontaneously hyperactive [26] and relatively lean since they appear to be less responsive to high-fat diet than C57 mice [27]. However, the development of diet-induced obesity in these two strains has not been formally compared. In this study, we developed a mathematical model predicting the change of the cell-size distribution as a function of the epididymal fat pad mass to analyze quantitatively the dynamic characteristics that depend on genetics and/or diet. The model of adipose tissue growth describes how many new cells are formed, how each cell grows depending on its size, and how lipid turnover leads to size fluctuations that cause a spreading in the cell-size distribution. As the epididymal fat pad mass increases, the cell-size distribution changes in a systematic manner depending on both genetics and diet. Comparing experimental results with the theoretical growth model, we found that hypertrophy is strongly correlated with diet. Hyperplasia, on the other hand, is dependent on genetics. Diet-induced changes in hyperplasia are strain-dependent, suggesting an interaction between diet and genetics.

## Results

### Effect of High-Fat Diet on Body Composition in FVB and C57 Mice

**Figure 1:**
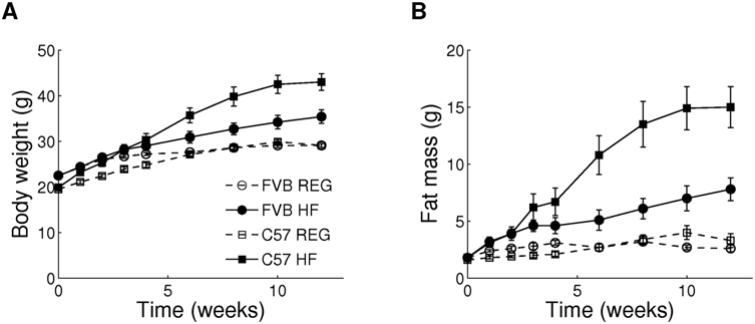
Changes in body composition of FVB and C57 mice under standard chow (REG) and high-fat (HF) conditions. (A) Body weight. (B) Fat mass. Body composition was measured at indicated time points in 8 mice per group using Echo 3-in-1 MRI analyzer. High-fat and control feeding were initiated at the age of 5 weeks. X-axis indicates weeks of controlled feeding. One of three independent experiments is shown. Values given are mean±SEM.

**Figure 2:**
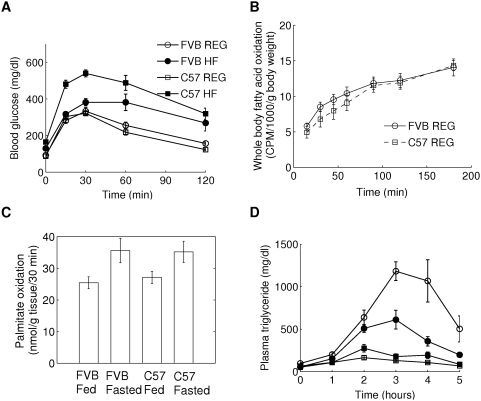
Physiological characteristics of FVB and C57 mice. (A) Glucose tolerance test was performed on week 10 of controlled feeding with chow and high-fat diet. Mice were fasted overnight and injected with glucose (2 mg/g, i.p.). Each group was represented by 8 mice. (B) Whole body oxidation of oleic acid was measured 10-week old male FVB ad C57 mice in non-fasted state (8 mice per group) as described in Gautam et al. [47]. (C) Oxidation in palmitic acids was measured in soleus muscle dissected from 10-week-old FVB and C57 mice maintained on chow diet (8 mice per group). Samples were collected at 8 am from randomly fed mice or mice fasted for 18 hours [methods in Toyoshima et al. [46]]. (D) Triglyceride clearance in FVB and C57 male mice after 11 weeks of controlled feeding with chow and high fat diet. Mice were fasted for 4 h and then given 400 *µ*l of peanut oil by a gavage. Plasma was collected hourly for 5 h from the tail vein for triglyceride measurement. Each group was represented by 8 mice.

At the beginning of the experiment (5 weeks of age), C57 mice were significantly lighter than FVB mice (Fig. 1A) due to a difference in lean mass, although total fat mass was not different (Fig. 1B). When mice were maintained on regular chow diet, the difference in body weight disappeared by the age of 11 weeks (week 6 of experiment, Fig. 1A). Under regular diet conditions, FVB and C57 mice maintained comparable fat mass throughout the whole course of the experiment (Fig. 1B). High-fat diet caused significant increase in body weight and fat mass in both strains; however, changes in body weight and fat mass were more dramatic in C57 mice. The C57 mice had twice as much fat after 12 weeks of high-fat feeding (Fig. 1B). The overall difference in total fat mass between FVB and C57 mice correlated with proportional differences in the amounts of epididymal (intra-abdominal), inguinal (subcutaneous), and brown fat (Table 1). Caloric intake and activity were comparable in FVB HF and C57 HF mice; however, FVB HF mice had higher resting and total oxygen consumption, and higher rectal temperature, suggesting that increased energy expenditure rather than reduced caloric intake was the reason for relative resistance to high-fat diet-induced obesity in the FVB mice. Interestingly, during the first 2 weeks of high-fat feeding, FVB and C57 mice showed comparable increase in total fat mass (Fig. 1B). C57 HF mice continued to increase fat mass rapidly until week 10 of the experiment, whereas FVB HF mice slowed down accumulation of fat around week 3. In C57 mice, high-fat feeding caused a gradual increase of both epididymal and inguinal fat pads; in contrast, in FVB mice, epididymal fat mass increased only slightly after 4 weeks on high-fat feeding, while inguinal fat pad continued to increase in size throughout the course of experiment (Fig. S1). High-fat feeding caused significant increase in blood glucose and insulin levels in both FVB and C57 mice (Table 1). Insulin levels and glucose intolerance were higher in C57 HF mice than in FVB HF mice, suggesting more severe insulin resistance (Fig. 2A). Consistent with previous reports [23],[24], C57 REG mice showed reduced serum triglyceride levels, compared with FVB REG mice with no difference in FFA (Table 1). This was not due to higher fat utilization, since respiratory exchange ratio (Table 1) and the rates of fatty acid oxidation measured *in vivo* (Fig. 2B) and in isolated skeletal muscle (Fig. 2C) were comparable in FVB REG and C57 REG mice. More likely, lower serum triglycerides in C57 REG mice were caused by much more efficient clearance of circulating triglycerides as suggested by triglyceride clearance test (Fig. 2D). High-fat feeding reduced circulating triglyceride levels in both FVB and C57 mice and improved triglyceride clearance in the latter strain (Table 1 and Fig. 2D). Both strains showed comparable reduction of respiratory exchange ratio, suggesting comparable increase of fatty acid utilization under high-fat diet condition (Table 1). Taken together, these data suggest that the ability to efficiently clear triglyceride from circulation may contribute to the high capacity of fat accumulation in C57 mice.

**Table 1:** Characteristics of FVB and C57 mice after 12 weeks of high-fat and control feeding.

	FVB REG^a^	FVB HF^a^	C57 REG^a^	C57 HF^a^	Strain^b^	Diet^b^	Interaction^b^
Body weight (g)	29.1±0.4	35.4±1.4	29.1±0.4	43.0±1.8	0.003	<0.001	0.003
Epididymal fat (g)	0.47±0.03	1.23±0.11	0.67±0.08	2.00±0.21	<0.001	<0.001	0.032
Inguinal fat (g)	0.34±0.02	0.82±0.13	0.45±0.04	1.92±0.27	0.003	<0.001	<0.003
Brown fat (g)	0.14±0.01	0.27±0.04	0.14±0.01	0.55±0.08	0.003	<0.001	0.003
Food intake (kcal/mouse/day)	16.6±1.0	18.0±1.3	13.2±0.8	17.7±0.8	0.07	0.007	ns
Total oxygen consumption (ml/g)	9.5±0.2	11.6±0.3	9.0±0.2	9.4±0.2	<0.001	<0.001	ns
Resting oxygen consumption (ml/g)	8.3±0.2	9.7±0.3	7.3±0.2	8.1±0.3	<0.001	<0.001	0.003
Respiratory exchange ratio	0.86±0.01	0.75±0.01	0.87±0.01	0.72±0.00	ns	<0.001	0.006
Total activity (beam brake/min)	265±5	190±17	345±41	201±16	ns	<0.001	ns
Ambulating activity (beam brake/min)	105±3	53±10	144±25	38±5	ns	<0.002	ns
Rectal temperature (°C)	36.0±0.2	36.6±0.2	35.4±0.2	35.5±0.2	<0.001	0.057	ns
Serum triglyceride (mg/dl)	190±25	95±24	89±16	64±6	0.002	0.004	ns
Free fatty acids (mM)	0.22±0.03	0.22±0.01	0.26±0.04	0.27±0.05	ns	ns	ns
Blood glucose (mg/dl)	187±16	225±11	209±11	247±15	ns	0.009	ns
Serum insulin (mg/dl)	1.1±0.1	1.9±0.3	2.2±0.8	4.9±1.2	0.011	0.033	ns
Triglyceride clearance (AUG^c^)	3238±476	1651±160	489±44	742±98	<0.001	0.011	<0.001
Glucose tolerance (AUG^d^)	28633±1372	39560±3217	25947±1212	52176±2892	0.045	<0.001	0.003

Values given are mean±SEM.

a n = 8.

b Two way ANOVA.

c Area Under Graph in Fig. 2D.

d Area Under Graph in Fig. 2A.

### Hypertrophy and Hyperplasia with Fat Mass Increase

**Figure 3:**
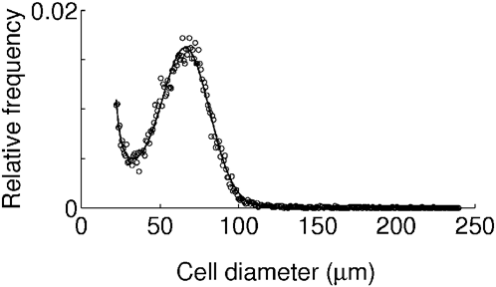
Normalized cell-size distribution in epididymal fat pad. One typical example of five-week-old control FVB mice is plotted. Circles indicate measured data points, while the line represents a fitting curve that is the sum of one Gaussian and two exponential functions.

**Figure 4:**
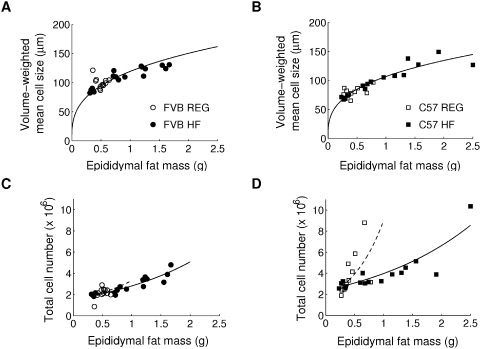
Size and number increase of epididymal fat cells with respect to epididymal fat pad mass. (A) and (B): the correlation between epididymal fat pad mass 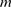 and volume-weighted mean cell size 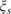 is fitted with a power law 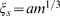 with (A) 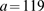 and (B) 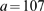, which is plotted with solid lines. (C) and (D): the correlation between epididymal fat pad mass 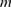 and total cell number 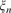 is fitted into an exponential function 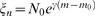 with initial fat pad mass 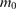, initial cell number 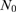, and cell-number increasing rate 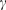, plotted with dashed lines (chow diet) and solid lines (high-fat diet). Fitted parameter values are summarized in Table 2.

To test the underlying mechanism of different rate of fat accumulation in epididymal fat of FVB and C57 mice, we measured mass and cell-size distribution in tissue samples of epididymal fat collected at 0, 2, 4, and 12 weeks of controlled feeding (Fig. 3). Since histological analysis does not allow accurate determination of adipocyte cell size, which will be discussed later, we measured cell size distribution using a Coulter Counter and estimated volume-weighted mean cell size and total cell number of epididymal fat pad from these measurements, which had similar values in other mouse study [5]. Strong correlations were observed between fat pad mass and volume-weighted mean cell size, and between fat pad mass and total cell number, regardless of strain and diet difference (Fig. 4). The first correlation gave a scaling relation, 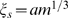, between fat pad mass, 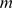, and volume-weighted mean cell size, 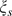 (Figs. 4A and 4B). In addition, an exponential relation was found between fat pad mass, 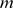, and total cell number, 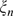: 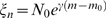 where the initial fat pad mass, 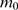, was obtained from control mice; and the initial cell number, 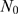, and the rate of increase of cell number in fat pad mass, 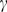, were estimated from data (Figs. 4C and 4D; Table 2). The initial cell number, 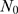, in C57 mice was larger than the initial cell number in FVB mice (Table 2; Figs. S2C and S2D). As the fat pad mass increases, the total cell number increases. The rate of increase of cell number, 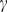, was larger under regular diet than under high-fat diet, a tendency more evident in C57 mice (Figs. 4C and 4D; Table 2), suggesting a genetic difference. The ratios of 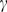 between the results of regular and high-fat diets are 1.42 and 3.22 for FVB and C57 mice, respectively (Table 2). This may indicate an interaction between genetics and diet on the increase of cell number. Note that we also observed similar results with body weight and fat mass, since the three quantities (epididymal fat pad mass, fat mass, and body weight) are correlated with each other. However, the results with epididymal fat pad mass were the best fits: The mean square deviation between data and fit in Figs. 4A–D was 9.73, 7.94, 3.56×10^5^, and 5.54×10^6^, respectively; the result with body weight was 11.82, 15.35, 3.65×10^5^, and 4.82×10^6^; the result with fat mass was 8.58, 8.61, 4.28×10^5^, and 6.11×10^6^.

**Table 2:** Parameter values of adipose tissue growth model.

	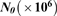 a	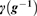 a	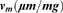 b	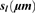 b	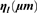 b	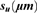 b	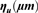 b	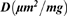 b
FVB REG	1.92±0.08 (1.91)	0.802±0.292 (0.841)	0.100±0.047	36±5	13±4	116±53	62±22	0.395±0.164
FVB HF	1.92±0.08 (1.91)	0.573±0.052 (0.591)	0.057±0.019	37±5	11±4	156±47	65±23	0.245±0.092
C57 REG	2.74±0.21 (2.75)	1.629±0.308 (1.650)	0.074±0.028	39±5	12±4	144±57	62±23	0.354±0.090
C57 HF	2.74±0.21 (2.75)	0.508±0.066 (0.512)	0.038±0.011	37±4	12±4	181±46	61±23	0.197±0.053

Values given are mean±SD.

a Parameters related to hyperplasia. Maximum-likelihood values shown in parentheses were used as the initial cell number 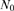 and the cell-number increasing rate 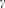 in the model.

b Parameters related to hypertrophy. The values are obtained from the average result of four ensembles; each ensemble corresponding to one control mouse has a different initial cell-size distribution for the model evolution. Note that the average cost (or error) 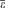, obtained from each ensemble, is considered with its Boltzmann factor 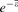 as a weight for the ensemble average.

### Genetic and Dietary Effects on Adipose Tissue Growth

**Figure 5:**
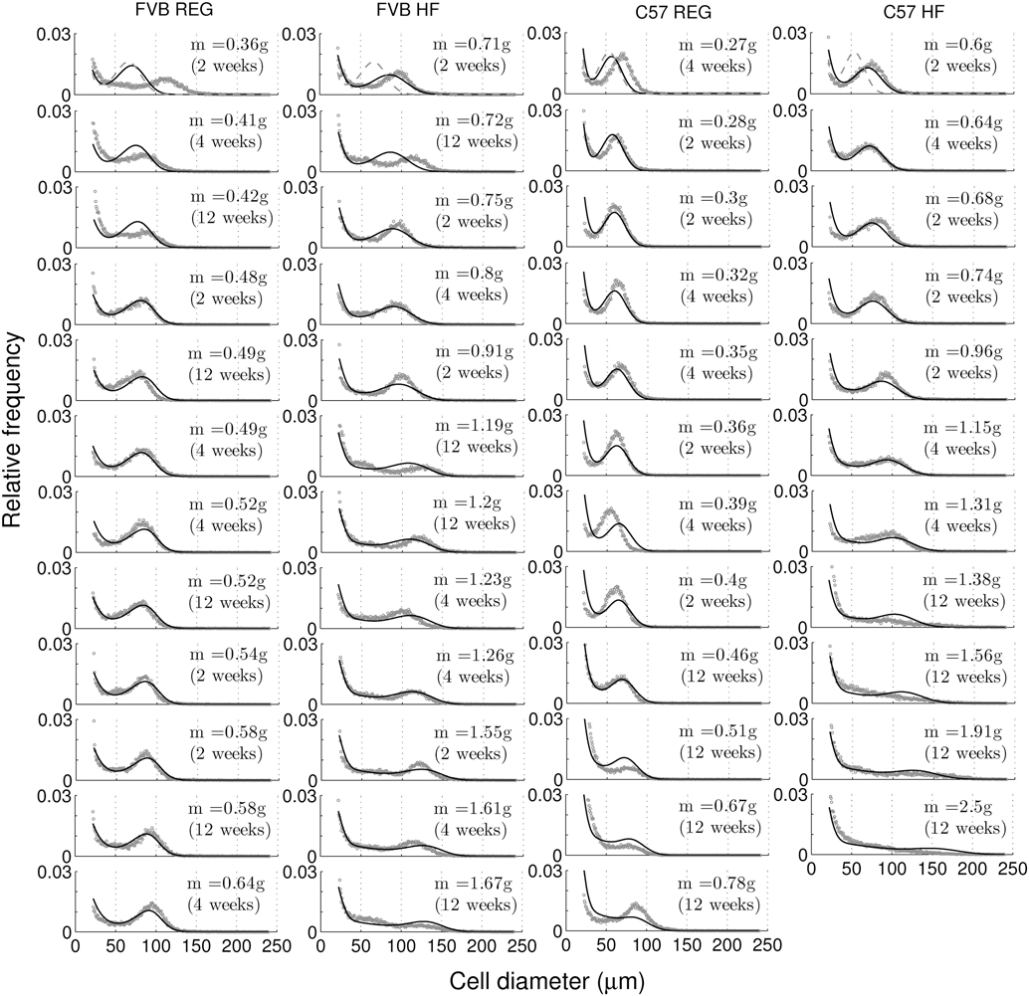
Changes of cell-size distributions with the mass increase of epididymal fat pad under chow and high-fat diets. Initial cell-size distributions of five-week-old control mice are given at the first row of each column with dashed lines. Dots show experimental results. Note that the results are ordered with epididymal fat pad mass, not with the chronological time (weeks of controlled feeding) shown in parentheses. Solid lines represent the normalized cell-size distributions corresponding to the given epididymal fat pad mass, predicted by the adipose tissue growth model.

**Figure 6:**
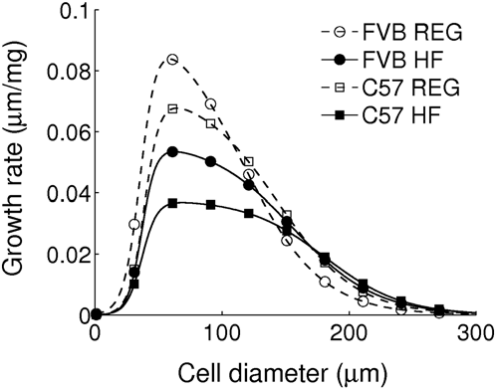
Size-dependent growth rate 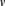 for the increment of epididymal fat pad mass. Here the mean parameter values in Table 2 are used for this plot. Symbols are shown to aid comparison between lines.

These two strong correlations, between fat pad mass and hypertrophy, and between fat pad mass and hyperplasia, suggest that the increase in adipose tissue can be described as a systematic growth process with respect to fat pad mass increase. We arranged cell-size distributions sorted with respect to epididymal fat pad mass (Fig. 5). Remarkably, the adipose tissue growth model in Eq. (1) describes the evolving pattern of cell-size distributions with respect to the fat pad mass increase. The model fitted experimental cell-size distributions quantitatively, despite the fact that all distributions are cross-sectional data obtained from individual animals. The different parameter values in the model, which fit each individual cell-size distribution from both strains and both diet regimens, gave quantitative differences in the epididymal adipose tissue growth process between strains and between diets (Table 2). First, the maximal size-dependent growth rate, 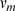, and the rate of cell-size fluctuations due to lipid turn over, 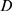, demonstrated a diet-induced difference, and a smaller strain-induced difference. Size-dependent growth and size fluctuations, which resulted in hypertrophy, appear to be regulated mainly by diet. Specifically, the size-dependent growth moved the large cell mode of the cell-size distributions in Fig. 5 to larger sizes, and the lipid turnover fluctuations increased the spread of the distribution around the large cell mode. It is important to note that the results must be carefully interpreted because every rate is a rate per unit fat pad mass increase, not per unit time increase. Second, the geometrical parameters (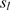, 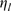 and 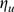), which determine the shape of the size-dependent growth rates, had essentially the same values regardless of the diet and strain difference, except for 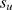. Therefore, the lower critical size, which gives the size initializing cell size-dependent growth, and two scale parameters could be fixed at *s_l_* = 37 *µ*m, *η_l_* = 12 *µ*m, and *η_u_* = 63 *µ*m, respectively. On the other hand, the upper critical size limiting cell size-dependent growth of big cells depended on diet; under the high-fat feeding, this cutoff size shifted to a larger size (Fig. 6). Under high-fat diet, the changes of parameters (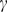, 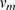, and 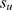) enlarge the lipid-storage capacity of fat tissues through both hyperplasia and hypertrophy. Lower serum triglycerides in the high-fat diet condition (Table 2 and Fig. 2D) may be correlated with the increasing lipid storage in enlarged fat cells because no significant difference in fatty acid oxidation was found as suggested by no difference in respiratory exchange ratio (Table 1).

## Discussion

Our central finding is that hyperplasia and hypertrophy of adipose cells in the epididymal fat pad is a function of the fat pad mass, even though it may take individual animals different time periods to reach a given fat pad mass. Therefore, adipose tissue growth, represented as changes of the cell-size distribution, can be systematically modeled as a growth process with respect to fat pad mass increase; this may reflect a correlation between fat pad mass and the secretion of adipokines and other signaling molecules controlling adipose tissue growth. Accordingly, it should be noted that the rates in our model are not the usual rates per unit time increase but the rates per unit mass increase. Thus, several rates (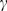, 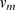, and 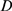) in the model had larger values for animals on a chow diet than for those on a high-fat diet. However, if these rates were converted to the usual rates per unit time increase, they had larger values for the high-fat diet, because it takes less time for a unit increase in the fat pad mass from larger, and more numerous, cells on a high-fat diet than for an increase of the same magnitude from smaller, and fewer, cells on a chow diet (Fig. S1). It has been suggested that when obesity progresses, hypertrophy of adipose cells occurs early, and then triggers hyperplasia [8]. Our study showed that new cell recruitment increases exponentially as fat pad mass increases. Hypertrophy of adipose cells is the main contributor to fat pad mass increase, whereas hyperplasia does not contribute much to this increase because it occurs in small cells that have a much smaller volume of fat stored. Therefore, our model naturally embodies the concept that hyperplasia is affected by the hypertrophic growth of cells. On the other hand, it has been reported that hyperplasia of adipose cells occurs only at early development stages; hence, no new cell recruitment would be expected at late stages even under obesogenic conditions [6],[7]. It may be the case that the age of the animals in our study (6 weeks old) allows the occurrence of hyperplasia.

The model developed here may give microscopic insights into the size-dependent growth of adipose cells that cannot be addressed by static cross-sectional studies. For example, we found the following specific properties of size-dependent cell growth: the lower critical size, 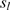, initializing lipid accumulation, did not depend on diet in the two mouse strains, whereas the upper critical size, 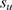, limiting cell growth from reaching an extraordinary size, was elevated on a high-fat diet. This size-dependence of cell growth is a testable hypothesis. Next, the cell-size fluctuation parameter, 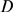, was different between regular and high-fat diets; it is larger under high-fat diet, when it is transformed to units appropriate for per unit time change instead of unit fat pad mass change. Thus, the random process for fat cells to release and take up fat occurs more actively under a high-fat diet than under a regular diet. It may be of interest to see if these results can be generalized to other strains and organisms.

Compared with the studies observing a single peak in cell-size distributions of fat cells [11], [15]–[17],[28], we have observed bimodal cell-size distributions as reported by others [12], [13], [29]–[32]. Most studies [12],[13],[31],[32] observing the bimodality used the Coulter Counter technology which has several advantages to assess the entire distribution of cell sizes [31]: First, the analyzed cells can be proven to be authentic adipose cells based on morphology and flotation; second, the volume of each cell is assessed regardless of shape and free of the artifacts of off-center sectioning as is the rule rather than the exception using histological approaches; finally, sufficient numbers of particles can be counted and sized to provide statistically significant complex curves. In contrast, microscopic methods for histology may not observe small cells due to the influence of microscope magnification [30], small sample number, and sampling bias. However, when the Coulter Counter is used, non-adipocyte contamination may contribute to the cell-size distribution especially at small sizes, although our minimal cell diameter, 22 *µ*m, is above the possible contamination ranges, 10 to 20 *µ*m, mentioned by Mersmann and MacNeil [31]. To be certain, we again analyzed the modified data using only cell-size distributions above 35 *µ*m diameter with the model, and reached the same conclusions (data not shown). The nadir in the cell-size distribution (Fig. 3) may separate two cell populations. DeMartinis and Francendese defined the small cells, with diameter smaller than 35 *µ*m, as “very small fat cells” [29]. Based on our model, these cells have negligible size-dependent growth, because their size is smaller than the lower critical size, *s_l_* = 37 *µ*m. Therefore, the size-dependent growth mechanism can naturally explain the origin of bimodality in the cell-size distribution of fat cells. Cells with size only above 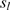 can grow with the size-dependent manner, but cells with size below 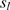 can randomly grow with the size-fluctuation through lipid turnover. This separation causes the cell accumulation below the size, 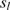, which gives the lower peak in cell-size distributions. This cell population may serve as a potential reservoir for mature adipose cells. Their maturation process may be interpreted as follows: The fat cells reaching the critical size, 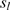, by random size fluctuations, then, can grow with a size-dependent growth mechanism. As mentioned above, the size fluctuation occurs more actively under a high-fat diet; therefore, the reservoir can accelerate the maturation process under the stimulating condition.

In the tissue growth model, we included the recruitment of new cells and the growth of existing cells, but not the death of old cells, because the model was consistent with the data without the apoptosis of adipose cells. This result is also consistent with a study observing that epididymal fat tissue of C57BL/6 mice does not show significant cell death before 12 weeks under a high-fat diet [33]. However, extended high-fat diet finally induces apoptosis of fat cells [33]. Furthermore, one recent study has reported that human fat cells turn over on a ten-year time scale [7]. Our model, therefore, still needs enhancement to be more generally applied in various conditions. Cell death should be considered and the diet dependence of the model parameters should be formally incorporated. In this study, we focused on one fat depot, epididymal fat, with several reasons: 1) the weight of epididymal fat pads can be accessed more accurately than the weight of inguinal fat pads due to the ease of dissection; 2) the morphology of adipose cells in epididymal fat is more homogeneous than in inguinal fat which contains a lot of brown adipose tissue-like cells, particularly in mice resistant to diet-induced obesity; and 3) the difference between the genotypes was more evident in growth of epididymal fat, which reaches a plateau at 4 weeks in FVB mice, but continues to grow in C57 mice throughout the course of experiment, in contrast to the inguinal fat shows sustained growth in both strains (Fig. S1). Although we have not measured the cell-size distribution of other fat depots, we measured the mass change of inguinal and brown fat depots, which shows similar pattern with epididymal fat depot (Fig. S1). Thus, it is of interest to apply the model to other fat depots that have functional differences [34],[35], and to other species such as human, which is left for future study. We expect the model can be applied to such diverse data sets simply by adjusting the model parameters, because the model contains general tissue growth mechanisms for the recruitment of new cells and their subsequent development.

Our data suggest that at least three factors may explain why C57 mice gain more fat than FVB mice do under high-fat diet: First, FVB mice have increased metabolic rate and increased rectal temperature, most likely due to the increased sympathetic tone. Although we did not detect significant differences in activity between the strains in our study, more comprehensive behavior measurements suggested that FVB mice are spontaneously hyperactive, compared with C57 mice [26]. They also have increased heart rate [36] and respond with hyperglycemia to a variety of treatments [37]. In addition, they are more responsive to stress associated with restraint and fasting [38]. All these data taken together suggest that the activity of sympathetic nervous system increased more in FVB mice than the activity in C57 mice. Second, compared with the FVB mice, C57 mice clear circulating triglycerides more efficiently [23],[24], which at least in part could be attributed to higher serum lipase activity [23] and higher capacity to store triglycerides in the liver [23],[24] and the adipose tissue (shown in this study). Although molecular mechanisms of triglyceride clearance are not fully understood, adipose tissue clearly contributes to clear triglycerides because the ability to clear circulating triglycerides is impaired in lipoatrophic mice [23]. In particular, it has been reported that high-fat diet enhanced triglyceride clearance [39], which may be related to the induction of lipoprotein lipase activity in the adipose tissue [40]. Finally, compared with FVB mice, C57 mice showed greater recruitment of small adipose cells, particularly under high-fat diet. It has been suggested that new adipose cells can arise from progenitor cells which reside within the adult white fat depots [41],[42], and from other sources such as bone marrow-derived circulating progenitor cells [43]. Recruitment of both types of progenitors has been shown to be stimulated by high-fat diet [41],[43]. It is possible that greater recruitment of smaller fat cells in C57 mice might be caused by a higher pool of precursor cells or thier higher intrinsic capacity for adipocyte differentiation. However, *in vitro* mesenchymal stem cell, isolated from the outer ear of FVB and C57 mice, differentiate into adipose cells equally well [44]. The attempt to compare differentiation of bone marrow stromal cell from FVB and C57 mice into adipose cells was not conclusive due to the very low yield and poor proliferative capacity of the cells isolated from C57 mice [45]; however, bone marrow does not appear to be the major source of new fat cells at least in mice [43]. Our model suggests the difference between genotypes in the recruitment of small adipose cells might be fat pad autonomous, but the molecular mechanism underlying this difference is unclear. Fat pad is a complex organ containing a variety of different cell types, including mature adipose, preadipose and vascular cells, nerves, macrophages, and fibroblasts. The number of adipocyte precursors and their proliferation in response to external signals varies between fat depots [35]. Further studies would be needed to determine how genotype specific interaction between different cell type and secreted factors may affect the rate of adipocyte recruitment to the specific fat depots.

In summary, we have derived a mathematical model describing the growth of adipose tissue with cell-number and cell-size increases as a function of epididymal fat pad mass. Based on this dynamic model, we examined the effects of genetics and diet on adipose tissue growth. Comparing the cell-size distributions from two strains and two diets, we concluded that cell size change depends on diet, and cell number change depends on genetics and diet, as well as their interaction.

## Materials and Methods

### Animals

All procedures were approved by the Animal Care and Use Committee of the National Institute of Diabetes and Digestive and Kidney Diseases. Male FVB and C57 mice were obtained from The Jackson Laboratory (Bar Harbor, ME). Mice were reared four per cage on a 12-h light/dark cycle (lights on 06:00–18:00). At the age of 5 weeks, mice of each strain were split into 2 groups. Half of the mice were fed regular chow NIH-07 diet (hereafter REG; Zeigler Brothers, Inc., Gardners, PA), containing 4.08 kcal/g (11% calories from fat, 62% from carbohydrates and 26% from protein). The other half was fed high-fat diet, F3282 (hereafter HF; Bio-Serv, Frenchtown, NJ), containing 5.45 kcal/g (59% fat, 26% carbohydrate, and 15% protein). Water and diets were provided *ad libitum*. Five independent experiments were conducted, each using 4 groups of mice: FVB REG, FVB HF, C57 REG and C57 HF. In three experiments, mice were maintained on controlled diets for 12 weeks and used for body composition analysis, physiological characterization , and cell size distribution. Two additional sets of mice were euthanized after 2 weeks and 4 weeks of high-fat and control feeding for cell-size distribution only.

### In Vivo Experiments

Body composition, food intake, metabolic rate, glucose tolerance, triglyceride clearance, and fatty acid oxidation in isolated soleus muscle were measured as described previously [46]. Whole body fatty acid oxidation was measured as described in Gautam et al. [47]. Blood for biochemical assays was obtained from the tail vein in the non-fasted state. Glucose levels were measured using Glucometer Elite (Bayer, Elkhart, IN). Serum insulin was assayed using radioimmunoassay (Linco Research, St. Charles, MO). Serum triglycerides, cholesterol (Thermo DMA, Louisville, CO) and free fatty acid (FFA) (Roche Applied Science, Indianapolis, IN) were measured according to the manufacturers procedures.

### Measurement of Cell Size in Epididymal Fat

Cell-size distribution in epididymal fat was measured after 2, 4, and 12 weeks of high-fat and control feeding using Beckman Coulter Multisizer III as previously described [9]. Briefly, 20–30 mg of fat tissue were sampled from the midsection, by dissection and then removing the sample for fixation from the center of the cut epididymal fat. Tissue samples were immediately fixed in osmium tetroxide [48], incubated in a water bath at 37°C for 48 h, and then adipose cell size was determined by a Beckman Coulter Multisizer III with a 400 *µ*m aperture. The range of cell sizes that can effectively be measured using this aperture is 20–240 *µ*m. The instrument was set to count 6,000 particles, and the fixed-cell suspension was diluted so that coincident counting was <10%. After collection of pulse sizes, the data were expressed as particle diameters and displayed as histograms of counts against diameter using linear bins and a linear scale for the x-axis (Fig. 3). Cell-size distribution was measured in four samples from each group, except for the C57 mice after 4-week high-fat diet exposure, which had only three available samples. A sample was taken from each fat pad and processed separately. Each sample was then counted at least twice. The curves from the two samples are then averaged, but only after examining the reproducibility between the two samples.

### Adipose Tissue Growth Model

The cell-size distribution includes all the information related to cell sizes in a tissue and its changes give a statistical view of the detailed growth process of each cell. To examine adipose tissue growth in terms of underlying microscopic processes, we consider a mathematical model quantifying the processes that change the cell-size distribution. The model can predict how many new cells are formed and how cells with different sizes grow as fat pad mass increases. The cell-number density 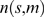 of a certain size 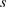 (diameter) at a given fat pad mass 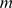 is the specific quantity to be considered. We consider how this cell-size distribution 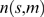 changes with an incremental change in fat pad mass 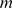. The evolution of the cell-size distribution with fat pad mass can be modeled by a partial differential equation,
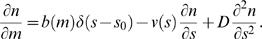
(1)This equation comprises three general components of the adipose tissue growth process. First, we assume that new cell recruitment occurs only at the minimal cell size 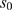 observed, which is mathematically expressed as the delta function 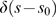. The recruitment rate 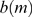 with respect to fat pad mass 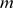 is given by the exponential function,
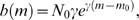
(2)where 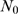 is the initial total cell number at a given initial fat pad mass 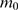, and 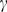 is the rate of increase in cell number per unit change in fat pad mass. The change of total cell number is the recruitment rate of new cells if cell death is negligible; we found no need to include apoptosis at any cell size to fit these experimental data. Therefore, this recruitment rate can be directly obtained from the experimental result using the relation 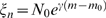 between total cell number 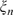 and fat pad mass 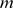 by differentiating the function 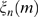 with respect to 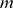. Second, there is cell-size dependent cell growth. After maturation of adipose cells to a specific size, they may be able to accumulate fat, causing hypertrophy. In addition to this limiting growth rate of small adipose cells, there may be also an upper growth limit because large adipose cells cannot grow indefinitely by this growth process, though they may attain larger sizes by size fluctuations caused by lipid turnover. The rise and fall of cell-growth rate depending on cell size can be described with the general functional form multiplying two sigmoidal functions,
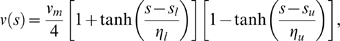
(3)where 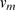 represents the maximal growth rate; 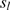 and 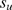 are the lower and upper critical sizes, respectively, which give the half-maximal growth rate; 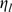 and 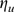 give their scale (Fig. 6). Finally, the last term in Eq. (1) represents cell-size fluctuations with the constant rate, 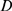, which reflect lipid turnover randomly occurring in adipose cells. This lipid turnover is the only growth mechanism for large cells above the upper critical size 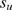. Generally, the size-dependent growth of cells moves their size distribution to larger sizes, while size fluctuations spread the size distribution.

### Quantitation of Hypertrophy

The volume-weighted mean cell size was used as a measure of hypertrophy,
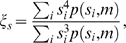
(4)where 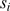 is the cell size of the 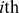 bin and 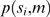 is the relative frequency corresponding to the size 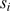 at a given fat pad mass 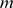. This measure is meaningful from the functional point of view that the lipid storage capacity is proportional to cell volume. Note that the volume-weighted mean cell size gives an average cell size weighted by the lipid-storage capacity of large cells in the upper peak of the bimodal cell-size distribution (Fig. 3). In contrast, the usual number-weighted mean cell size, 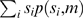, may give a smaller average cell size due to the considerable contribution of small cells in the lower peak of the bimodal cell-size distribution. Clearly, the volume-weighted mean cell size is a better index of lipid-storage capacity.

### Quantitation of Hyperplasia

Total cell number is a direct measure reflecting hyperplasia. In this study, epididymal fat pad mass as well as cell-size distributions were measured. Therefore, the total cell number in the epididymal fat pad could be estimated from the relation 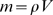 between fat mass 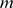 and volume 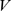. Here we used pure trioleine density *ρ* = 0.915 g/ml as the density of adipose cells since the density is similar to the actual fat pad density [49]. The total fat volume 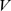 is 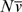 where 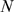 is the total cell number and 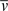 is the average cell volume, which could be calculated from the cell-size distribution. Therefore the total cell number 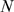, or hyperplasia index 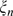, is
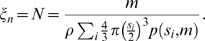
(5)


### Optimization Method

To optimize model parameters 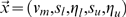 so that they can closely describe the evolution of cell-size distribution in experiment, we used the minimization of a “cost function” which quantifies the deviation between the model and experimental results. To define the cost function, the normalized cell-size distribution 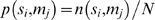 at a given fat mass 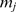 was compared with simulation data 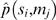 with a parameter set 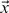:
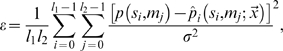
(6)where 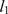 is the total number of cell size bins and 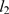 is the total number of given fat mass. The scale 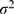 of the cost function was calculated from the intrinsic fluctuation of experimental data, which can be defined as the squared deviation between the measured cell-size distribution 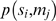 and a smooth fitting function 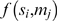:
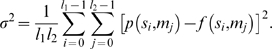
(7)This intrinsic fluctuation is numerically about 10 percent of the squared deviation between experimental and model data. As the fitting function in Fig. 3, we used a sum of two exponentials and a Gaussian,
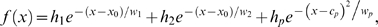
(8)a form that has been used to fit adipose cell-size distributions [9]. These parameter fits were performed using the nonlinear curve-fitting routine in MATLAB R2007a (Natick, MA, USA). For the optimization process, we specifically used the parallel tempering Monte-Carlo method to find the global minimum of the cost function [50]. We used 10 uniformly spaced values (0.1 to 1) for the tempering parameter 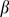 and ran ten chains in parallel with the updating probability 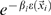. At every 20 steps, a pair of adjacent simulations on ten tempering parameters were randomly chosen and their parameter states were exchanged with probability 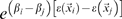. After equilibrium, 20,000 iterations were used with the fixed tempering parameter 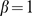 to estimate the optimal parameter values and their standard errors.

We also used this method to estimate the initial total cell number, 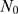, and its rate of increase, 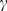, from the relation between fat mass and total cell number (Figs. 4C and 4D). In the minimization between the fitting function 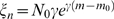 and experimental data, we used a constraint that the initial cell number is equal regardless of diet conditions, i.e., regular and high-fat diets. The average fat mass of four control mice before regular and high-fat diets was used as the initial fat mass, 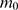, which is 0.34 g and 0.29 g for FVB and C57 mice, respectively. We estimated the uncertainties, 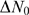 and 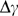, by propagating the 10 percent statistical fluctuations observed in the experimental data.

### Numerical Solution of Partial Differential Equation

We solved the following discrete version of our model, given as a continuous partial differential equation in Eq. (1):
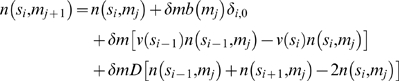
(9)with mass interval *δm* = 0.1 mg and size interval *δs* = 0.73 *µ*m.

## Supporting Information

Figure S1 Changes in epididymal (A, B), inguinal (C, D), and brown (E, F) fat pad mass of FVB (A, C, E) and C57 (B, D, F) mice under chow and high-fat diet conditions. X-axes indicate weeks of controlled feeding initiated at the age of 5 weeks old. Y-axes in panel (A–D) shows combined weight of right and left fat pads. Each time point has four sample per group with exception of week 12 (eight samples per group) and 4 week C57 HFD (three samples per group). Lines are drawn for guide.
(0.09 MB TIF)

Figure S2 Changes in epididymal fat pad of FVB and C57 mice under chow and high-fat diet. (A) and (B) Volume-weighted mean cell size. (C) and (D) Total cell number. X-axes indicate weeks of controlled feeding initiated at the age of 5 weeks old. Each time point has four samples at the time point, four weeks (three available samples). Lines are drawn for guide.
(0.07 MB TIF)
